# Production of transgenic *Paulownia tomentosa* (Thunb.) steud. using chitosan nanoparticles to express antimicrobial genes resistant to bacterial infection 

**DOI:** 10.22099/mbrc.2019.35331.1454

**Published:** 2020-06

**Authors:** Eman Tawfik Hussien

**Affiliations:** Lecturer of Genetics and Genetic Engineering, Faculty of Science, Helwan University, Egypt

**Keywords:** Chitosan nanoparticle, Erwinia carotovora, thionin genes transformation

## Abstract

*Paulownia tomentosa* (Thunb.) Steud. is a very important hard woody plant, an extremely fast-growing tree and produce timber. Therefore, there is a demand to produce transgenic *Paulownia* plant resistant to bacterial infection. Microbial infection (especially bacterial one) is serious sever and cause a loss in plant productivity as they bear upon the character and amount of plant product. Two phytopathogenic bacteria were chosen to consider their effect on *Paulownia tomentosa*. These two bacterial species were *Erwinia carotovora* and *Pseudomonas aeruginosa*. Two thionin genes (AT1G12660 and AT1G12663) were selected. They produce antimicrobial peptides to resist this bacterial infection. Chitosan nanoparticle is a novel technology in genetic transformation into plant tissues. Chitosan nanoparticles were used in a ratio of 1:1 with the plasmid DNA carrying thionin genes independently. Characterization for chitosan nanoparticles was applied to determine the conditions of genetic transformation. The new transgenic *P. tomentosa* lines produced are partially resistant to these two bacterial infections compared to non-transgenic lines. The inhibitory percentage in the transgenic lines ranged from 8 to 21% wherein the non-transgenic the inhibitory percentage of *P. tomentosa* leaves ranged from 53-24%. Likewise, it is noticed that is *Paulownia tomentosa* less infectious than *Erwinia carotovora.* In conclusion, I recommend using chitosan nanoparticle is an excellent way for gene transformation into plant tissues. Also, manipulate the idea of using thionin as antimicrobial genes to resist bacterial infection for different plant species.

## INTRODUCTION

*Paulownia tomentosa* Steud. (Also called royal *Paulownia*, empress or princess tree) is a species native to China. Today it has spread all over the world and increased in popularity and is grown in many countries. *Paulownia* belongs to family Paulowniaceae (Scrophulariaceae). It is known as an economically important multipurpose tree genus due to its quick development and short-rotation for production of timber. *Paulownia* is seen as a woody biofuel crop [1, 2]. Investigaotors enumerated the value of *Paulownia* as a short-rotation woody crop plants, afforestation, mine site reclamation, ornamental use, the bark has been used in Chinese herbal medicine as a component remedies for some infectious diseases, used to make furniture, musical instruments, flooring and finally the wood of *Paulownia* is soft, lightweight with excellent machining and finishing properties [3-5]. *Paulownia* *tomentosa, also* shows cytotoxic activity against several human cancer cell lines and inhibit the effects of human cholinesterase, butyrylcholinesterase, and bacterial neuraminidases. 

Microbial infection affects plant, resulting in losses and decreasing the quality and safety of agricultural products. *Erwinia carotovora* as a rod-shaped gram-negative phytopathogenic bacterium and deadliest pathogen affects productivity of plants. It causes bacterial rot for plant leaves [6]. *Pseudomonas aeruginosa* as a fluorescent bacterium was isolated from diseased tissues [7]. *P. aeruginosa* is gram-negative, aerobic rod belonging to the bacterial family Pseudomonadaceae [8]. It is commonly found in soil and water. It occurs regularly along the surfaces of plants and occasionally along the surfaces of an animal body. They are one of the few groups of bacteria that are true pathogens of plants and cause leaf blight. Thionins are a family of pathogen-related proteins (PR) with antimicrobial activity. There are many sorts of thionins found in seeds, stems, roots, or leaves of different plant tissues [9].

Chitosan nanoparticle is a non-viral polymer nanoparticle used for transferring genetic material to the nucleus. Chitosan is a linear polysaccharide, composed of Glucosamine and N-acetyl Glucosamine units linked by β (1-4) Glycosidic bonds and a partially deacetylated product of the natural polysaccharide chitin. Chitosan is obtained by deacetylation of chitin under alkaline conditions. The size and charge distribution (charge density) are very important factors for the in vitro and in vivo performance of polymeric delivery systems. According to other studies, the polymeric gene delivery systems (with a positive charge) complexes with the DNA with a negative charge). This increase the encapsulation efficiency and improve the uptake of DNA into the cells via the interaction of the negatively charged cell membrane [10].

This study report for the first-time optimization procedure of transformation of thionin genes using chitosan nanoparticles and subsequent regeneration of transgenic plantlets in which the integration of transgenes is proven. Besides, this study aimed to produce *Paulownia* *tomentosa* resistant to bacterial infection (*Erwinia carotovora* and *Pseudomonas aeruginosa*).

## MATERIALS AND METHODS

**Plant materials:** *Paulownia tomentosa* as woody plant species was kindly supported by agriculture center for genetic engineering and biotechnology (ACGEB). It was cultured on free MS media for propagation. 

**Obtaining of Thionin Genes: **The total genomic DNA of *Arabidopsis thaliana* was extracted using Edward’s protocol described previously [11]. For the PCR reaction 50 ng of template DNA was used for each 25 μl reaction. Each reaction mixture contained 12.5 μl of 2X master mix (Biolene), 0.25 μltaqpolymerase (Biolene), 1 μl of each forward and reverse primer (50nmole/base) and complete up to 25 μl by sterile dd H_2_O. The thionin primers were designed using snap gene® (2.3.3) software and their sequences were as follows: Thio60F: 5’-GCT GAA TTC ATG GAG GAC AAA AGA-3’, Thio60R: 5’-GCT AAG CTT TCA TAG ACT AAA ATC AAT-3’; where Thio63F: 5’-GCT GAA TTC ATG TTG GTG GCA G-3’ and Thio63R: 5’-GCT AAG CTT AGT TTT TCT TGG TAC-3’. PCR reaction for each gene was performed for 40 cycles as follows: 30 min at 94^o^C, 30 min at 64^o^V and 1 min at 72^o^C. The two thionin genes to be determined were AT1G12660 (*Thio-60*) and AT1G12663 (*Thio-63*) genes and they are found on the chromosome one of Arabidopsis thaliana. PCR products of the isolated two thionin genes were run on 0.8% (w/v) agarose gel. The amplified PCR products were purified using the GeneJET™ PCR Purification Kit (Thermo K0701).

**Preparation of Modified Plasmid with Thionin Genes: **The purified *Thio-60 *and* Thio-63* product were ligated separately to pMiniT vector (NEB® PCR Cloning Kit, #E1202S. The kit was supplied with NEB 10-beta competent *E.coli* (NEB #C3019)) following the manual instruction to obtain a modified pMiniT with *thionin* genes separately. 

**Confirmation of Bacterial Transformation (Colony PCR): **This was applied to distinguish between recombinant and non-recombinant colonies; and performed using colony PCR. Bacteria with modified plasmid were grown on LB agar plates with 100 µg/L ampicillin. Separate colonies obtained from bacterial transformation were used as a template for PCR reaction. The primers, protocol and conditions for this reaction were as mentioned previously during *thionin* genes detection from *Arabidopsis thaliana*. After that, apply the product on 1.2% gel electrophoresis. 

**Chitosan nanoparticle transformation Chitosan nanoparticle characterization Determination of degree of deacetylation of Chitosan: **One form of chitosan nanoparticle was used for transformation. There are many methods for determination of degree of deacetylation of chitosan nanoparticles; one of them is titration method. Czechowska-Biskupand et al., illustrated this method as follows: Dried chitosan (0.2 g) was dissolved in 20 cm^3^ 0.1M hydrochloric acid and 25cm^3^ [12]. After 30 min continuous stirring, the succeeded portion of deionized water (25cm^3^) was added and stirring continued for 30 minutes. When chitosan was completely dissolved, the solution was titrated with a 0.1 mol·dm^-3^ sodium hydroxide solution using an automatic burette (0.01cm^3^ accuracy). Degree of deacetylation (DA or DD) of chitosan was calculated utilizing formula:

$\mathrm{DA\%}=2.03\left(\frac{\mathrm{V2}-\mathrm{V1}}{m+0.0042\left(\mathrm{V2}-\mathrm{V1}\right)}\right)$

Where: m – weight of sample, V1, V2- volumes of 0.1 mol·dm-3 sodium hydroxide solution corresponding to the deflection points, 2.03 – coefficients resulting from the molecular weight of chitin monomer units, 0.0042- coefficients resulting from the difference between molecular weights of chitin and chitosan monomer units.

**UV-Visible Spectra Measurements: **UV-visible spectra were recorded using a JASCO V-630 UV-visible spectrophotometer (serial: C285061148) (using Spectra Measurement software) to confirm the chitosan nanoparticle formation. 

**Size and zeta potential of chitosan nanoparticle from Zeta-Sizer: **The size and potential of chitosan nanoparticles were measured in high-performance particle Zetasizer HPPS-5001 (Malvern, the UK). Each sample was analyzed in triplicate at 25°C at a scattering angle of 90°C. Pure water was utilized as a reference for dispersing medium. The results are given as the average particle size obtained from the analysis of three different batches, each of them measured three times. Particle size measurements were made in cuvettes at 37 °C by non-invasive back scatter, with dynamic light scattering detected at an angle of 173°C. In chitosan suspensions the buffer used was 25 mM acetic acid (pH 3.0) and for complex preparations a mixture of 25 mM acetic acid pH ≈ 3.0 and 10 mM Tris-HCl (pH 8.0) was used in the same volume ratio of chitosan per pDNA molecule (N/P ratio dependent). 

Zeta potential measurements were also performed at 37°C using a combination of laser Doppler velocimetry and phase analysis light scattering (PALS). The measured electrophoretic mobility was converted into zeta potential values using the Smoluchowski approximation. Zeta potential and hydrodynamic diameters are expressed as the mean standard deviation of three independent measurements [13, 14]. 

**Preparation of Chitosan-DNA nanoparticles (CS/pDNA):** Mansouri et al., identified the method of CS/DNA formation as follows: Chitosan (CS) was dissolved in 25 mM acetic acid, which was then adjusted to pH 5.5 at a final concentration of 1% (stock solution). The stock solution was then diluted with distilled water to concentrations of 0.08%. The CS and the modified pMiniT were first incubated in a water bath at 55^o^C for 15 min. Then CS – DNA complexes were prepared by adding CS (0.08%) to an equal volume of a pDNA solution (50mg/ml) followed by immediately intense stirring on a vortex mixer for 1 min. *(Note: for transformation with chitosan, the plasmid was dissolved in 50 mM sod. sulfate)*

**Transmission Electron Microscopy: **Carbon coated 400 mesh copper grids were put over one drop of the complex (CS/pDNA) and left to stand for 1.5 min. The grid was stained with one drop of filtered solution containing 2% uranyl acetate for 1.5 min, and any excess uranyl acetate was removed with filter paper [16]. The grids were allowed to dry for a further 10 min and then examined with a transmission electron microscope in the Regional Center for Mycology and Biotechnology, Al-Azhar University. 

**Transformation of Chitosan/pDNA Into Plant Tissue: **The method of transfection of chitosan nanoparticles into plant tissue was developed by [17, 18]: *P. tomentosa *nodal segments of 0.5-1cm were incubated in Eppendorf tube containing 300 µl of CS/pDNA (1:1) mix for 30 min at room temperature. After that the explants were transferred on to MS medium containing (2 mg/l BA and 1 mg/l kin) hormones and 100 µg/L ampicillin then incubated at 25 ± 1 °C for 4 weeks to regenerate plants. 

**Molecular Analysis of Transgenic Plants: **Total genomic DNA was extracted from the leaves of transgenic and non-transgenic *P. tomentosa* plants using cetyl-trimethyl ammonium bromide (CTAB) method [19]. DNA fragments of *thio-60 *and* thio-63* transgenes were amplified using the total genomic DNA as templates by PCR with a pair of primers as described previously with the same PCR program conditions. 

**Pathogenicity Bioassay: **This was performed via bacterial-resistance assay of transgenic plants. The antimicrobial activity of the thionin genes in the transgenic plants was tested against two different bacterial strains (*Erwinia carotovora* and *Pseudomonas aeruginosa*). Pathogenicity bioassay was performed via bacterial suspension infecting plant’s leaves. 

**Disease Resistance Assay of Whole** ***In Vitro*** **Plants: **This is done as described previously with some modification [20]. Fifty microlitres of LB broth culture of *E. carotovora *and *P. aeruginosa* were inoculated within the plants jar (3 weeks old) grown on 100 ml MS medium and incubated at 25^o^C with 16h light/8h dark regime. Pictures were taken 2 weeks after inoculation.

**Statistical Analysis: **The statistical analysis of results obtained were analyzed using Minitab 19. It was performed to obtain mean average of reading, significance and F-value. 

## RESULTS AND DISCUSSION

In this study, transformation of both *Thio-60* and *Thio-63* genes were conducted using chitosan nanoparticles. Commercial *Paulownia *species were used: *Paulownia tomentosa*. Nodal cutting technique (micropropagation) of tissue culture was used for this transformation. The transgenic plants exhibited enhanced resistance to *Erwinia carotovora* and *Pseudomonas aeruginosa* infection. Both thionin genes (*Thio-60* and *Thio-63*) were isolated from *Arabidopsis thaliana* plant. They were manipulated using PCR with their specific primers, respectively, and detected on 1.2% agarose gel. Then, the PCR product for each gene independently was inserted into pMini-T vector. After that, the modified plasmids were transformed into competent 10-beta *E. coli*. The colony PCR for confirmation of both thionin transformations into 10-beta *E. coli* for amplification. *Thio-60* was detected nearly at 640 bp and *Thio-63* at 480 bp. 

**Figure 1:**
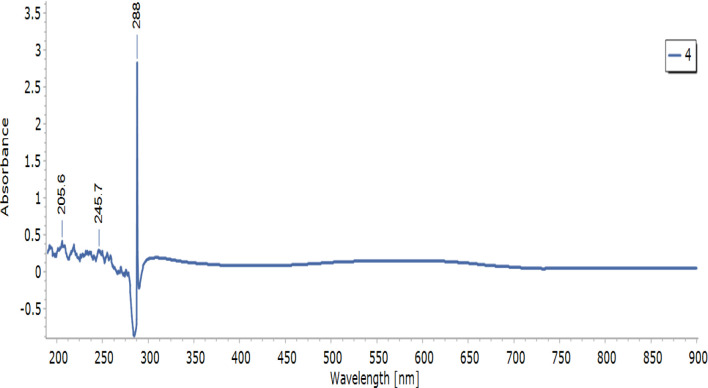
UV-visible spectrum of chitosan nanoparticle

The chitosan nanoparticle used in this study was characterized in the Table 1. This describes DD, morphology, pH, TEM measurements, UV-visible measurements, zeta size and potential (Fig. 1).

**Table 1:** Description and characters of the chitosan complex with modified plasmids

**Character**	**Morphology**	**pH**	**Average DD**	**Combined**	**UV**	**Particle**	**Zeta**
				Particle	Spectroscoy	size “nm”	potential
				Diameter	“nm”	Of free	“mV”
				“nm”		Chitosan	
**Chitosan**	Powder	4.40	51.23±5.765	P60:	288	97.4±8.9	39.5±9.27
**description**	Whitish			195.98±2.24			
	yellow			P63:			
				193.72±2.66			

**Figure 2:**
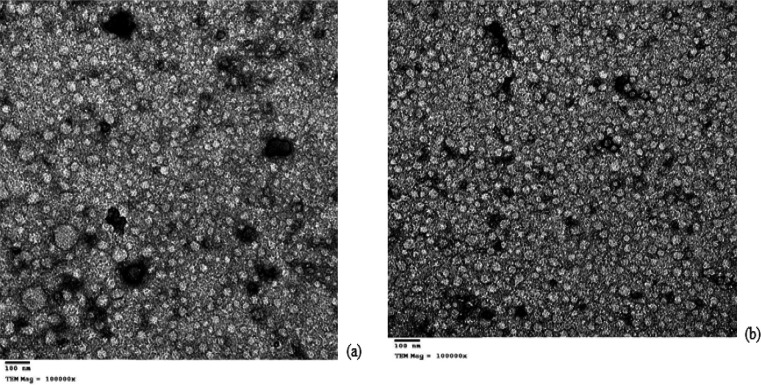
Transmission electron microscopy of chitosan nanoparticle binding with pMini-T carrying both thionin genes: (a) CS/P60, (b) CS/P63. Image scales: 100 nm

Also, TEM images illustrated the complex of chitosan nanoparticles with modified plasmids carrying thionin genes separately (Fig. 2). The estimated degree of deacetylation was 51.23 and the deacetylation degree of chitin, which is the content of glucosamine, is higher than about 50%, it becomes soluble in an aqueous acidic medium such as acetic acid [10]. 

The picture of CS/pDNA complex was photographed under TEM according to methods of Kiang *et al.,* photographed chitosan with plasmid containing human embryonic kidney cells (HEK293) [16], and Hallaj-Nezhadietel *et al.,* which photographed pUMVC3-hIL12 loaded chitosan nanoparticles in Colon Carcinoma Cells [21]. Up till now few researches included application of chitosan in the plant and the work in this field is too narrow and Tawfik *et al*., who photographed chitosan nanoparticle binding with pEGAD plasmid [18]. In this study the potential of chitosan nanoparticles was 39.5 mV. This agrees with Ing *et al.,* (2012) who estimated the potential of chitosan nanoparticles, which ranged from 33 to 54 mV [22]. 

**Figure 3:**
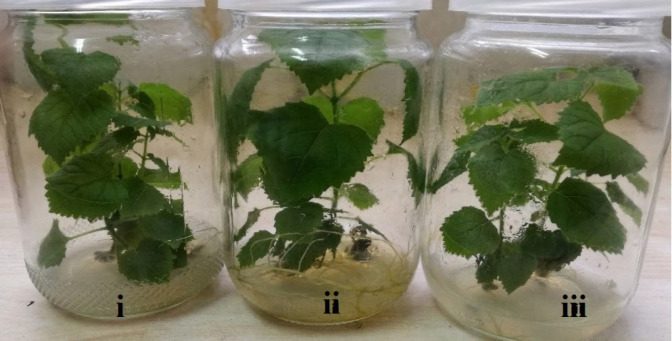
Non-transgenic and transgenic *Paulownia tomentosa*. i: non-transgenic, ii: transgenic with *Thio-60*, iii: transgenic with *Thio-63*

After four weeks incubation at 25^o^C under light conditions (16 h light and 8 h dark), the regenerated shoots of the transgenic and non-transgenic species (*Paulownia tomentosa*) were fully grown as shown in the figure 3. Chitosan nanoparticle was previously utilized for gene transformation for different sources like animal cell lines and human cell lines [16, 21]. However, it is still limited in plant gene transformation used chitosan nanoparticle transformation into potato plant tissues [17, 18]. 

To confirm the transformation of thionin genes into *P. tomentosa*, PCR for both transgenic species were applied. There were two different bands were detected, about 640 bp for *Thio-60* and 480 bp for *Thio0-63*. Also, PCR for non-transgenic species was applied to confirm that both thionin genes were not found in them. 

**Figure 4:**
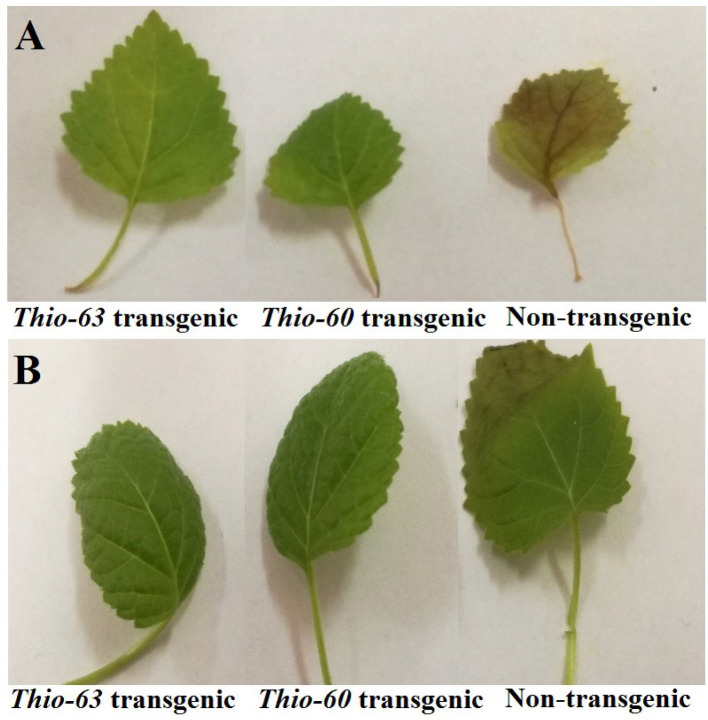
Leaves of transgenic and non-transgenic *Paulownia tomentosa* infected with *Erwinia carotovora* (A) and *Pseudomonas aeruginosa* (B)

In this bioassay, we use the bacterial suspension of two different bacterial pathogens (*Erwinia carotovora* and *Pseudomonas aeruginosa*) to infect the detached leaves of *Paulownia tomentosa* plant. *Erwinia carotovora* causes leaf rot while *Pseudomonas aeruginosa* causes leaf blight disease. The results showed a highly significant difference in response between the transgenic and non-transgenic lines of *P. toementosa* (Table 2, Fig. 4). There is a significant difference between the response of *P. tomentosa* expressing *Thio-60* and *Thio-63* inhibitory proteins. As antimicrobial peptides, the expressed *Thio-63* protein is more resistant to bacterial infection than *Thio-60* one. This occurs in both types of bacterial pathogen *Erwinia carotovora* and *Pseudomonas aeruginosa*. 

**Table 2:** Inhibition percentage of detached leaves of *P.* *tomentosa* infected with bacterial suspension

**Bacteria**	**Paulownia Transgene**	**Mean ± SD**	**F-value**
*Erwinia carotovora*	Non-transgenic	53.537 ± 2.969	259.05**
	*Thio-60* transgenic	21.273 ± 2.094	
	*Thio-63* transgenic	14.967 ± 1.293	
*Pseudomonas aeruginosa*	Non-transgenic	24.423 ± 0.841	325.56**
	*Thio-60* transgenic	13.143 ± 0.983	
	*Thio-63* transgenic	8.938 ± 0.315	

** highly significant P<0.001

The inhibitory effect of bacterial infection in case of *Pseudomonas aeruginosa *is less than *Erwinia carotovora*. This could be due to some resistant components inside the leaves of *P. tomentosa* and this is confirmed by the in vitro study of aqueous extracts of fresh *Paulownia* leaves have antimicrobial effects against *Pseudomonas aeruginosa* [5].

Thionins are believed to be involved in protection against plant pathogens, including bacteria and fungi, by working directly at the membrane. They are isolated from different plant parts (endosperm, leaf or root). Besides, they were identified in many plants like *Arabidopsis thaliana*, *Hordium vulgare* and *Triticum aestivum*. Approximately 100 individual thionin sequences have been identified in more than 15 different plant species [23, 24].

Thionin genes were previously described as an antimicrobial effect (resist fungal and bacterial infection). The expressed thionin proteins were demonstrated to be antifungal for many phytopathogenic fungi by many scientists who transform thionin genes into potato plant to resist some fungi like *Alternaria alternata, Rhizoctonia solani, Fusarium solani *and* Fusarium oxysporum* [17, 18]. As antibacterial, Cp-thionin II (47 aa) was identified from *Vigna unguiculata* cowpea seeds and is antibacterial against both gram-positive and gram-negative bacteria such as *Staphylococcus aureus*, *Escherichia coli*, and *Pseudomonas syringae* [25]. Also, both thionin and definsin displayed antimicrobial activities against *E. coli* and *Micrococcus luteus* [26].

Finally, it is recommended to use chitosan nanoparticles in genetic transformation into different plant tissues, and recirculate the idea of transforming different thionin genes which have antimicrobial effect into different plant species. 
